# The effect of endotoxin adsorber haemoperfusion on microcirculation in septic pigs

**DOI:** 10.1186/2197-425X-3-S1-A420

**Published:** 2015-10-01

**Authors:** Y-C Yeh, C-Y Wu, A Chao, W-S Chan, Y-J Cheng, W-Z Sun, T-Y Lin, NTUH Center of Microcirculation Medical Research (NCMMR)

**Affiliations:** Department of Anesthesiology, National Taiwan University Hospital, Taipei, Taiwan Province of China; Department of Anesthesiology, Far Eastern Memorial Hospital, New Taipei, Taiwan Province of China

## Introduction

Microcirculatory dysfunction plays an important role in sepsis-related multiple organ dysfunction.(1) Several studies has shown polymyxin B hemoperfusion has favorable effects on mean arterial pressure, vasopressor use, and mortality.(2) One rat sepsis study had found that microcirculation was better maintained in the polymyxin B hemoperfusion group.(3) However, the effects of polymyxin B hemoperfusion on the microcirculation of the intestinal mucosa, intestinal muscular-serosal layer, kidney, and liver were unknown. We used a fecal peritonitis-induced septic pig model to investigate the effect of polymyxin B perfusion on the microcirculation.

## Objectives

This animal study aimed to investigate the effect of polymyxin B-immobilized fiber column hemoperfusion on the microcirculation of multiple organs in septic pigs.

## Methods

**Figure 1:**
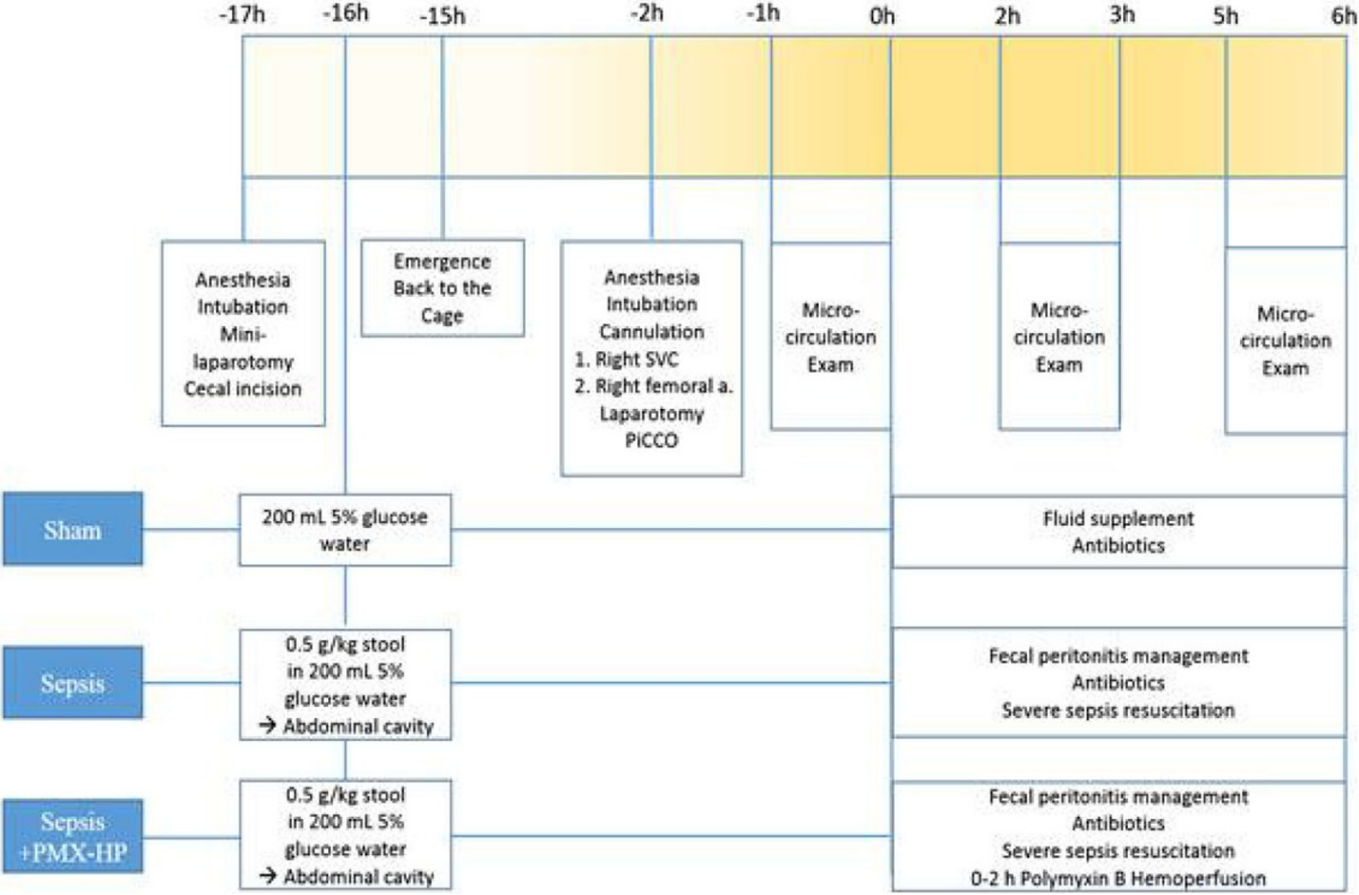
**Time course of the animal model.**

Eighteen male Lanyu pigs (Taitung Animal Propagation Station, Taiwan, body weight 25 ± 4 kg) will be investigated. This study was approved by the Institutional Animal Care and Use Committee. The animals were randomly assigned to the following 3 groups: 1 - Sham; 2 - Sepsis (fecal peritonitis model); and 3 - Sepsis + PMX-HP (fecal peritonitis model + polymyxin B-immobilized fiber column hemoperfusion). Time course of the animal model is shown in the Figure 1.

In the Sepsis and Sepsis + PNX-HP groups, 0.5 g/kg autologous feces and 200 mL 5% glucose were injected into the abdominal cavity. The hemodynamic parameter was monitored by the Pulse index Contour Continuous Cardiac Output system. A sidestream dark-field video microscope and a tissue oxygen monitor were used to investigate microcirculation.

## Results

12 pigs completed the study (4 for each group). The hemodynamic parameters were shown in the Table 1.

**Figure 2:**
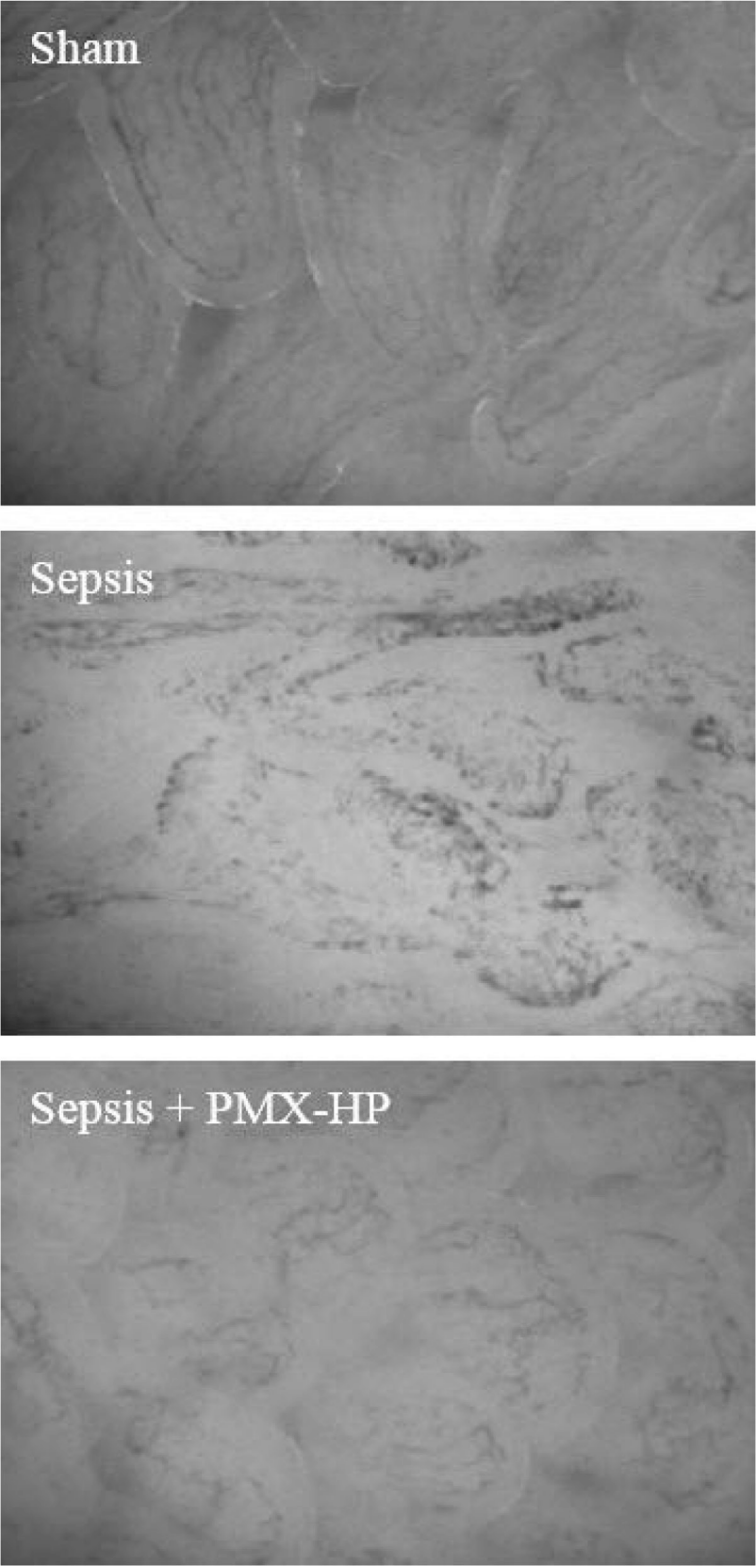
**Microcirculation of terminal ileal mucosa (6h).**

The perfused small vessel density (PSVD) of the terminal ileal mucosa in the septic pigs was less than the non-septic pigs at 0h (24.3 mm/mm^2^ vs. 30.0 mm/mm^2^, p = 0.005). The images of the microcirculation of the terminal ileal mucosa at 6h were shown in Figure 2.

**Table 1:** Hemodynamic parameters.

	Sham	Sepsis	Sepsis + PMX-HP
N	4	4	4
HR_0h (bpm)	132 (14)	160 (22)	174 (28)
HR_6h (bpm)	127 (49)	188 (3)	169 (13)
MAP_0h (mm Hg)	90 (12)	94 (20)	80 (26)
MAP_6h (mm Hg)	74 (8)	77 (18)	75 (5)
CI_0h (L/min/m2)	3.5 (0.7)	3.4 (1.3)	3.4 (0.4)
CI_0h (L/min/m2)	3.3 (0.6)	3.4 (0.3)	3.1 (0.7)
EVLWI_0h (mL/kg)	11.8 (2.6)	14.4 (2.7)	12.3 (1.9)
EVLWI_6h (mL/kg)	13.5 (2.4)	17.4 (4.7)	14.5 (4.6)

Table 2 represents that the PSVD of the terminal ileal mucosa at 6h in the Sepsis + PMX-HP group had a trend to be greater than the Sepsis group (25.7 mm/mm^2^ vs. 16.3 mm/mm^2^, p = 0.066).

**Table 2:** Microcirculation comparison.

		Sham	Sepsis	Sepsis + PMX-HP
Intestinal mucosa	PSVD_0h (mm/mm2)	30.0 (2.4)	25.2 (3.9)	23.5 (5.0)
	PSVD_6h (mm/mm2)	31.2 (2.2)	16.3 (11.7)	25.7 (5.1)
	StO2_0h (%)	54 (7)	41 (18)	41 (22)
	StO2_6h (%)	66 (2)	42 (16)	48 (6)
Kidney	PSVD_0h (mm/mm2)	35.1 (3.0)	26.9 (12.1)	24.0 (7.5)
	PSVD_6h (mm/mm2)	37.3 (2.0)	22.8 (17.6)	24.2 (4.3)
	StO2_0h (%)	69 (12)	58(8)	63 (11)
	StO2_6h (%)	74 (8)	63 (5)	66 (10)

**Figure 3:**
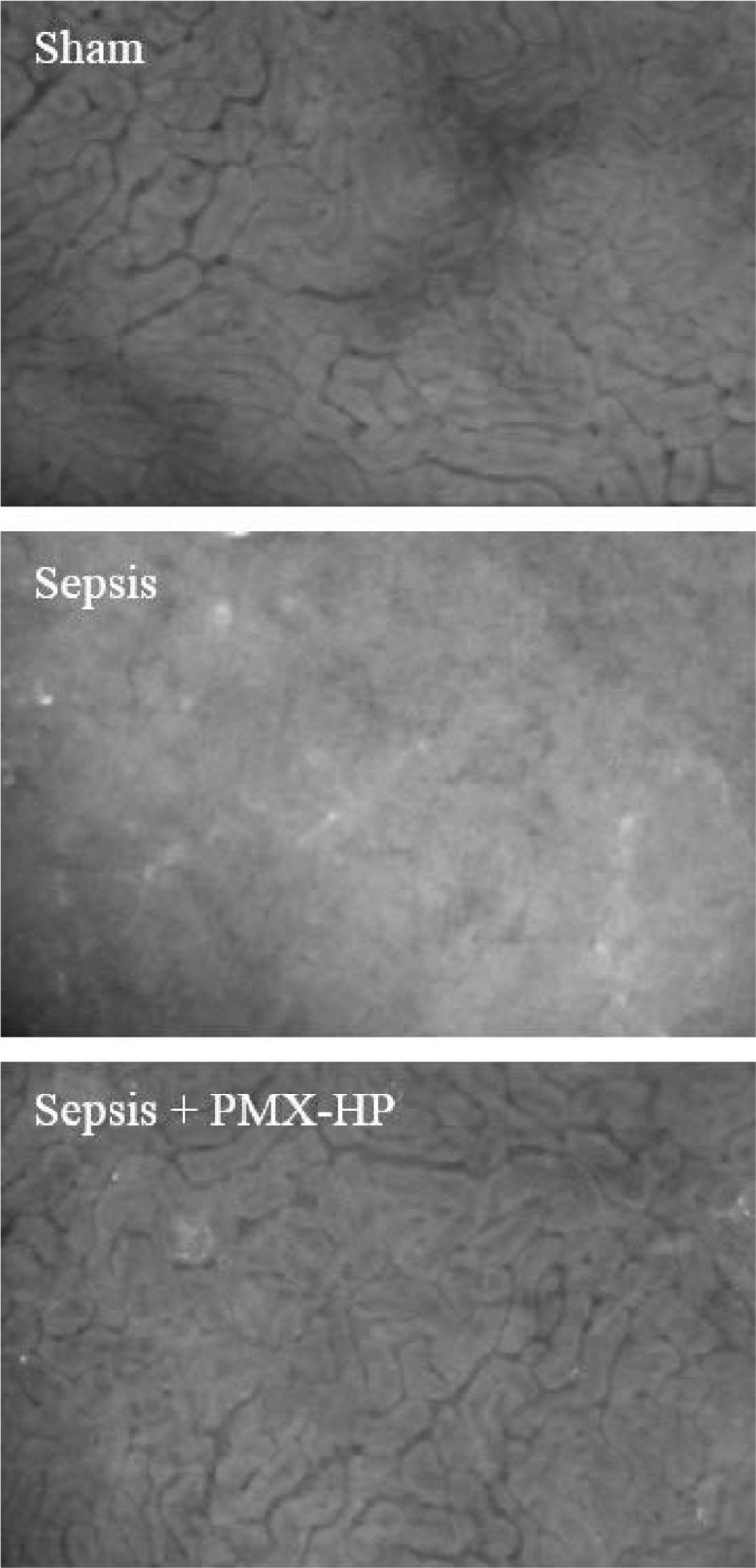
**Microcirculation of kidney surface (6h).**

The images of the microcirculation of the kidney surface at 6h were shown in Figure 3.

Table 3 represents the fact that the urine output in the Sepsis + PMX-HP group was greater than in the Sepsis group.

**Table 3:** Vasopressor, urine output, and laboratory results.

	Sham	Sepsis	Sepsis + PMX-HP
Norepinephrine_6h (mcg/kg/min)	0.09 (0.16)	0.62 (0.56)	0.33 (0.39)
Fluid therapy 0-6h (mL)	2212 (754)	2912 (999)	3262 (430)
Urine output 0-6h (mL)	191 (30)	131 (69)	256 (71)
Creatinine_0h (mg/dL)	1.3 (0.2)	1.6 (0.9)	1.5 (0.3)
Creatinine_6h (mg/dL)	1.3 (0.2)	2.0 (1.3)	1.5 (0.6)
ALT_0h (U/L)	24 (5)	33 (3)	27 (6)
ALT_6h (U/L)	20 (5)	32 (11)	22 (3)
Lactate_0h (mmol/L)	1.2 (0.5)	3.9 (2.5)	3.9 (1.9)
Lactate_6h (mmol/L)	1.6 (0.6)	4.2 (1.9)	3.8 (3.1)

## Conclusions

In summary, we found that polymyxin B hemoperfusion for septic pigs improves urine output and has a potential to attenuate the microcirculatory dysfunction of the terminal ileal mucosa.

## Grant Acknowledgment

Supported, in part, by research grant 103-FTN11 and NTUH.103-A125 from the Far Eastern Memorial Hospital and National Taiwan University Hospital Joint Research Program.
